# Glucose Dehydrogenases-Mediated Acclimation of an Important Rice Pest to Global Warming

**DOI:** 10.3390/ijms241210146

**Published:** 2023-06-14

**Authors:** Peng-Qi Quan, Jia-Rong Li, Xiang-Dong Liu

**Affiliations:** Department of Entomology, Nanjing Agricultural University, Nanjing 210095, China; 2018202043@njau.edu.cn (P.-Q.Q.); 2022102061@stu.njau.edu.cn (J.-R.L.)

**Keywords:** antioxidase, *Cnaphalocrocis medinalis*, global warming, glucose dehydrogenase, heat acclimation, reactive oxygen species, superoxide dismutase

## Abstract

Global warming is posing a threat to animals. As a large group of widely distributed poikilothermal animals, insects are liable to heat stress. How insects deal with heat stress is worth highlighting. Acclimation may improve the heat tolerance of insects, but the underlying mechanism remains vague. In this study, the high temperature of 39 °C was used to select the third instar larvae of the rice leaf folder *Cnaphalocrocis medinalis*, an important insect pest of rice, for successive generations to establish the heat-acclimated strain (HA39). The molecular mechanism of heat acclimation was explored using this strain. The HA39 larvae showed stronger tolerance to 43 °C than the unacclimated strain (HA27) persistently reared at 27 °C. The HA39 larvae upregulated a glucose dehydrogenase gene, *CmGMC10*, to decrease the reactive oxygen species (ROS) level and increase the survival rate under heat stress. The HA39 larvae maintained a higher activity of antioxidases than the HA27 when confronted with an exogenous oxidant. Heat acclimation decreased the H_2_O_2_ level in larvae under heat stress which was associated with the upregulation of *CmGMC10*. The rice leaf folder larvae may acclimate to global warming via upregulating *CmGMC10* to increase the activity of antioxidases and alleviate the oxidative damage of heat stress.

## 1. Introduction

Temperature crucially affects the growth, survival, and reproduction of insects [1,2]. Global warming causes a significant increase in average temperature and extremely high temperatures [3], which poses a greater threat to insects, a huge group of terrestrial ectotherms [4,5]. Exposure to 40 °C for 2 h led to the death of 100% *Bradysia odoriphaga* larvae within 5 days [6], and exposure to 42 °C for 5 d reduced the adult survival rate of the flour beetle *Tribolium castaneum* by approximately 30% and of pupae by 100% [5]. However, acclimation to a mild heat may increase the tolerance capacity of insects to an extremely high temperature [7] by inducing heat shock proteins and altering oxidoreductase activity [8,9,10,11].

Oxidoreductase catalyzes redox which is involved in basic metabolism. Oxidoreductases are closely related to the reproduction of insects. Glucose dehydrogenase (GDH) facilitates sperm uptake and release through the spermathecal ducts of the female *Drosophila melanogaster*, and GDH-mutant females store only one-third as much sperm as wild-type females [12]. The GDH-silenced small brown planthopper females laid 48.6% fewer eggs than the control females [13]. Oxidoreductase may affect insect immunity. The upregulated glucose–methanol–choline (GMC) oxidoreductase GMCβ genes increase the survival rate of the silkworm when infected with pathogens [14]. A high level of FAD-glucose dehydrogenase-specific activity is induced by abiotic implants (sterile latex) in the larvae of *Manduca sexta* [15]. Oxidoreductases are also related to biological and environmental stress pressed on organisms. Heat stress affects the metabolism of animals [16,17,18], which may be mediated by oxidoreductases.

Under environmental stress, a large number of reactive oxygen species (ROS) will be produced, which bring about oxidative damage to organisms. Multiple antioxidant enzymes in organisms inhibit the accumulation of ROS [19]. Superoxide dismutase (SOD) can protect cells from damage by oxygen radical generation in the livers of rats [20]. When whiteflies, *Bemisia tabaci*, were exposed to 4 °C and 40 °C, SOD activity was significantly increased [21]. In the citrus red mite *Panonychus citri*, the activities of SOD and glutathione-S-transferase (GST) were significantly increased when the mite was undergoing thermal stress [22].

RNA interference (RNAi) based on dsRNA is an economical, convenient, and powerful technique for exploring gene function. It has been widely used in Coleoptera, Hemiptera, Hymenoptera, etc. [23,24,25,26]. Although the interference efficiency using dsRNA is not very stable in Lepidoptera insects, depending on species and genes, the RNAi is still an alternative method and has been used well in the silkworm *Bombyx mori*, diamondback moth *Plutella xylostella*, and rice leaf folder *Cnaphalocrocis medinalis*, etc. [27,28,29,30,31]. The dsRNase in insects can degrade dsRNA, which decreases the interference efficiency of the target gene [32,33]. The *CmdsRNase* gene has been cloned in *C. medinalis*, and injection of dsCmCHS with dsCmdsRNase increased the interference efficiency of *CmCHS* by 27.17% [34]. Therefore, the improved RNAi will be used in Lepidoptera insects until a convenient gene editing technique is developed and becomes popular.

The rice leaf folder is a serious pest of rice in Asia whose populations have a migratory habit. In summer, populations migrate stepwise from southwest towards northeast China and then return in autumn [35]. This migratory habit can help adults avoid regional high temperatures in summer. However, the pest populations are unable to escape from global warming, which leads to the rise of the average temperature and extremely high temperatures. Although the rice leaf folder is liable to high temperatures [36,37], under the threat of global warming, the population density and outbreak frequency increased in the 21st century [38,39,40]. Previous studies found that the rice leaf folder larvae could generate a heat-acclimated strain via multigenerational selection at a high temperature [8,10,41], and heat acclimation-induced differentially expressed genes enriched in the GO term of oxidoreductase activity [10]. Therefore, here we hypothesized that heat acclimation altered the expression profile of oxidoreductase activity genes, which increased the antioxidant capacity of larvae to tolerate heat stress. In this study, the roles of two glucose dehydrogenase genes belonging to the GMC family, *CmGMC10* and *CmGMC38*, were studied using heat-acclimated and unacclimated strains, and we found that *CmGMC10* mediated the heat acclimation of larvae via acting on antioxidases and reducing ROS levels under heat stress. The results addressed the molecular mechanism of an insect’s ability to adapt to heat stress.

## 2. Results

### 2.1. Heat Tolerance of the Heat-Acclimated Larvae

**Figure 1:**
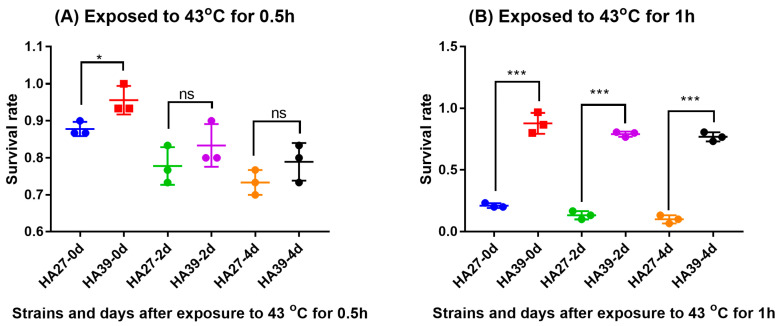
Survival rates of the heat-acclimated (HA39) and unacclimated (HA27) strains after exposure to 43 °C for 0.5 h (**A**) and 1h (**B**). * and *** indicate significant differences between HA27 and HA39 at *p* = 0.05 and 0.001 levels, respectively.

Heat acclimation significantly increased the tolerance of larvae to a high temperature which the larvae had not previously experienced. The survival rate of the HA39 was much higher than that of the HA27 when they were exposed to 43 °C for 0.5 h (t = 3.13, df = 4, *p* = 0.035; Figure 1A) and 1h (t = 13.338, df = 4, *p* < 0.0001; Figure 1B). Although the survival rates of larvae between HA27 and HA39 at 2 d (t = 1.250, df = 4, *p* = 0.279) and 4 d (t = 1.581, df = 4, *p* = 0.189) after exposure to 43 °C for 0.5 h were similar (Figure 1A), the survival rates of HA39 were still significantly higher than that of the HA27 at 2 d (t = 28.778, df = 4, *p* < 0.0001) and 4 d (t = 23.398, df = 4, *p* < 0.0001) after exposure to 43 °C for 1 h (Figure 1B).

### 2.2. Effect of Heat-Acclimation on Expression Levels of Oxidoreductase Genes

**Figure 2:**
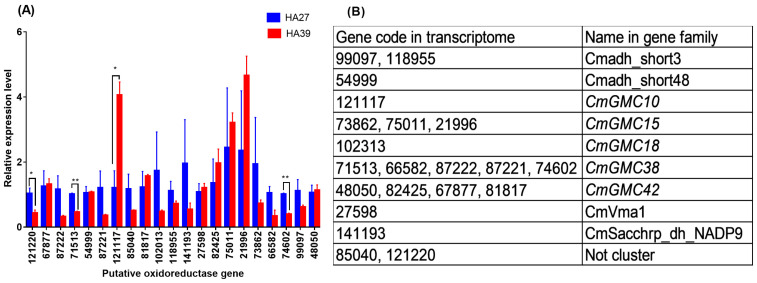
Relative expression levels of 21 putative oxidoreductase genes of the heat-acclimated (HA39) and unacclimated larvae (HA27) exposed to 41 °C for 1 h (**A**) and their classification in the gene family (**B**). * and ** indicate significant differences between HA27 and HA39 at the *p* = 0.05 and 0.01 levels, respectively.

When larvae were exposed to 41 °C for 1 h, 4 out of 21 putative oxidoreductase genes were differentially expressed between the HA27 and HA39 (Figure 2A), and only 121117 was significantly upregulated in the HA39, compared to the HA27 (t = 4.223, df = 4, *p* = 0.013), whereas the other three, 121220 (t = 2.978, df = 4, *p* = 0.041), 71513 (t = 9.441, df = 4, *p* = 0.001), and 74602 (t = 9.705, df = 4, *p* = 0.001), were significantly downregulated (Figure 2A). Based on the genome of *C. medinalis*, 14 putative genes were clustered into 5 GMC family genes, and 74602 and 71513 were the same gene (Figure 2B). 121117 and 71513 were clustered in *CmGMC10* and *CmGMC38*, respectively (Figure 2B). A partial sequence of *CmGMC10* and the full length of *CmGMC38* were cloned (Figure S2). Sequence alignment in NCBI showed that the two genes belong to the subfamily of glucose dehydrogenases in the GMC gene family.

### 2.3. Spatiotemporal Expression Patterns of Two Oxidoreductase Genes

**Figure 3:**
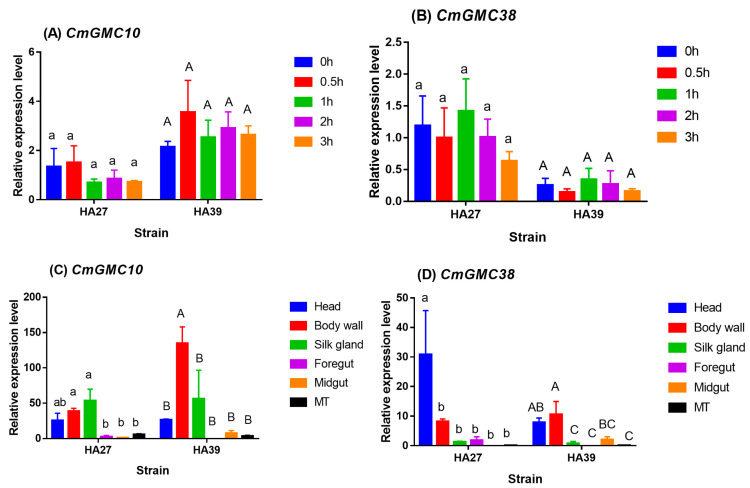
Spatiotemporal expression profiles of two oxidoreductase genes, *CmGMC10* and *CmGMC38*, of the HA27 and HA39 larvae. The relative expression levels of *CmGMC10* (**A**) and *CmGMC38* (**B**) in the HA27 and HA39 strains exposed to 39 °C for 0–3 h. The relative expression levels of *CmGMC10* (**C**) and *CmGMC38* (**D**) in different body parts of the HA27 and HA39 larvae. MT: Malpighian tubule. Different lowercase and uppercase letters indicate significant differences among different exposure durations or body parts of the HA27 and HA39 larvae, respectively.

The relative expression levels of *CmGMC10* of HA27 (*F_4, 10_* = 0.605, *p* = 0.668) and HA39 (*F_4, 10_* = 0.486, *p* = 0.746) were not significantly different among the larvae exposed to 39 °C for 0, 0.5, 1, 2, and 3 h (Figure 3A). Similarly, the relative expression levels of *CmGMC38* of HA27 (*F_4, 10_* = 0.523, *p* = 0.721) and HA39 (*F_4, 9_* = 0.512, *p* = 0.729) were also affected little by the exposure duration of 0–3 h at 39 °C (Figure 3B). However, these two oxidoreductase activity genes exhibited an obvious tissue-specific expression profile in the HA27 and HA39 larvae (Figure 3C,D). The *CmGMC10* gene was specifically expressed in the body wall of the HA39 larvae (*F_5, 12_* = 7.257, *p* = 0.002), whereas it was expressed mainly in the silk gland and body wall of the HA27 larvae (*F_5, 11_* = 5.938, *p* = 0.007; Figure 3C). The *CmGMC38* was expressed specifically in the head of HA27 larvae (*F_5, 11_* = 3.495, *p* = 0.039), and it was expressed mainly in the body wall and head of HA39 larvae (*F_5, 12_* = 5.129, *p* = 0.010; Figure 3D). The body wall and head-specific and stable expression profiles in the HA27 and HA39 imply that these two oxidoreductase genes might be associated with the heat acclimation of larvae because the body wall and head are the organs that first feel heat stress.

### 2.4. Effects of Knockdown of CmGMC10 and CmGMC38 on Heat Tolerance

**Figure 4:**
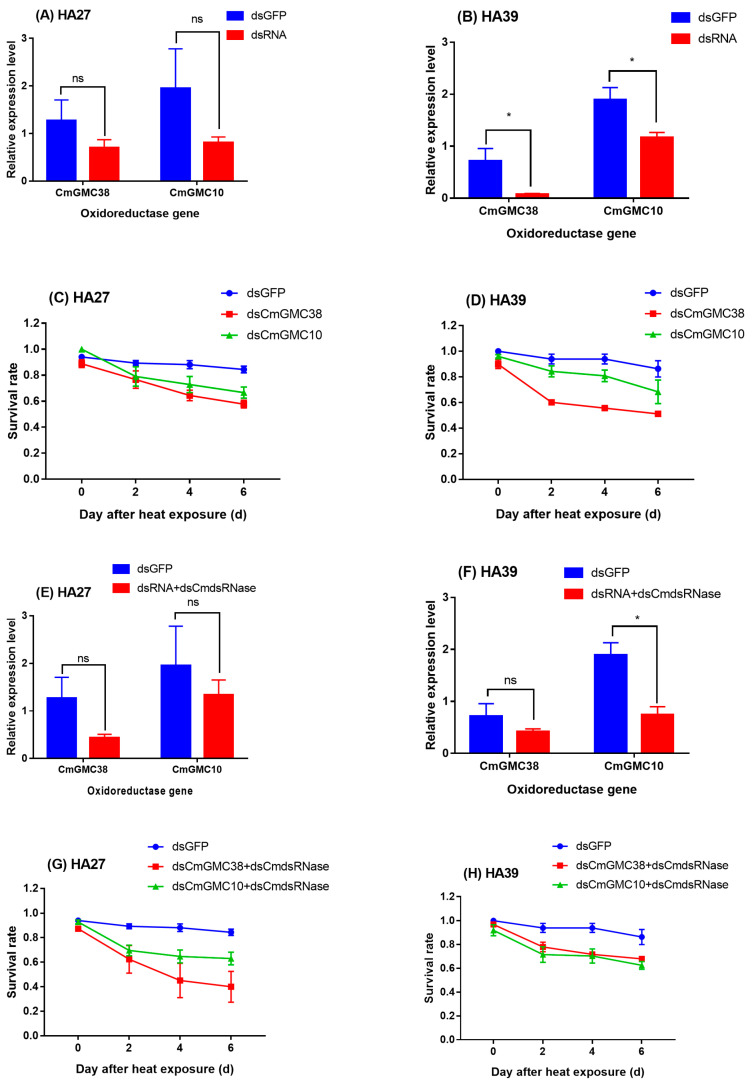
Knockdown of *CmGMC38* and *CmGMC10* using injection of dsRNA (**A**,**B**) and dsRNA+dsCmdsRNase (**E**,**F**) and its effect on the survival rates of the HA27 and HA39 larvae at 0–6 d after 3 h heat exposure to 39 °C. (**A**–**D**) Larvae injected with dsRNA and (**E**–**H**) larvae injected with dsRNA+dsCmdsRNase (1:1). * Indicates significant difference between the relative expression levels in the larvae injected with dsGFP and dsRNA or dsRNA+dsCmdsRNase. ns: not significant.

Injection of dsCmGMC10 and dsCmGMC38 into the HA27 and HA39 larvae decreased the expression levels of *CmGMC10* and *CmGMC38*, respectively (Figure 4A,B,E,F). Interference of *CmGMC38* or *CmGMC10* decreased the survival rates of both the HA27 (Figure 4C,G) and HA39 larvae (Figure 4D,H) after exposure to 39 °C for 3 h. The survival rates of HA27 larvae with knocked down *CmGMC38* by injection of dsCmGMC38 (*F*_2, 6_=6.251, *p* = 0.034) or dsCmdsRNase+dsCmGMC38 (*F*_2, 7_ = 7.381, *p* = 0.019) were significantly lower than larvae injected with dsGFP after heat exposure to 39 °C for 3h, whereas knockdown of *CmGMC10* did not affect the survival rate of HA27 (Figure 4C,G). Knockdown of *CmGMC38* using dsCmGMC38 decreased the survival rate of HA39 after heat exposure (*F*_2, 6_ = 29.859, *p* = 0.001; Figure 4D), and knockdown of *CmGMC10* using dsCmdsRNase+dsCmGMC10 also significantly decreased the survival rate of HA39 (*F*_2, 6_ = 8.330, *p* = 0.019; Figure 4H). The expression levels of *CmGMC10* and *CmGMC38* were associated with the heat tolerance of the larvae (Figure 4).

### 2.5. Enzyme Activity of Larvae after Knockdown of an Oxidoreductase Gene

**Figure 5:**
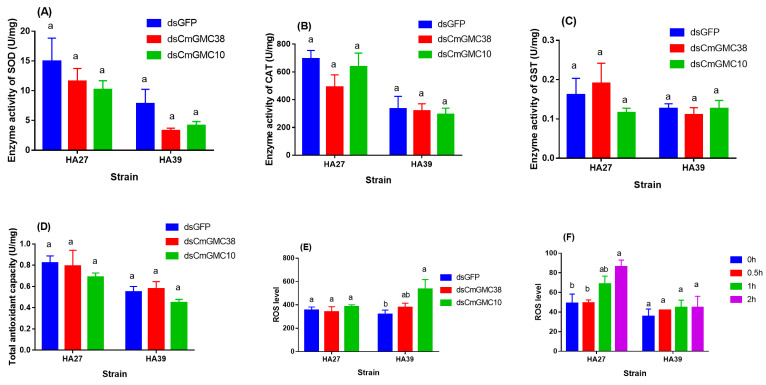
Enzyme activities of SOD (**A**), CAT (**B**), and GST (**C**), total antioxidant capacity (**D**), and ROS levels (**E**) of the HA27 and HA39 larvae exposed to 39 °C for 3 h after knockdown of *CmGMC10* or *CmGMC38* genes, and ROS levels of the HA27 and HA39 larvae exposed to 41 °C for 0–2 h (**F**). Different letters indicate significant differences among knockdown of *GFP*, *CmGMC10*, and *CmGMC38* of the HA27 and HA39 larvae. SOD: Superoxide dismutase, CAT: Catalase, GST: Glutathione transferase, ROS: Reactive oxygen species.

Knockdown of *CmGMC10* or *CmGMC38* did not significantly affect the enzyme activity of SOD, CAT, GST, and total antioxidant capacity (T-AOC) of the HA27 (SOD: *F_2, 15_* = 0.877, *p* = 0.436; CAT: *F_2, 14_* = 1.175, *p* = 0.337; GST: *F_2, 16_* = 1.073, *p* = 0.365; T-AOC: *F_2, 16_* = 0.618, *p* = 0.551) and HA39 (SOD: *F_2, 13_* = 2.121, *p* = 0.160; CAT: *F_2, 15_* = 0.089, *p* = 0.915; GST: *F_2, 15_* = 0.267, *p* = 0.770; T-AOC: *F_2, 15_* = 1.639, *p* = 0.227) larvae when exposed to 39 °C for 3 h, but these activities in the HA39 were lower than in the HA27 (Figure 5A–D). The ROS level in the HA27 larvae under heat exposure to 39 °C for 3 h was not significantly affected by the knockdown of *CmGMC38* or *CmGMC10* (*F_2, 7_* = 0.439, *p* = 0.662, Figure 5E), but the knockdown of *CmGMC10* resulted in a significant increase in the ROS level in the HA39 larvae under heat exposure (*F_2, 15_* = 4.694, *p* = 0.026, Figure 5E). When the HA27 and HA39 larvae without knockdown of these two genes were exposed to 41 °C for 0–2 h, the ROS levels in the HA27 were increased significantly as the exposure duration extended (*F_3, 10_* = 4.499, *p* = 0.03), but the ROS levels in the HA39 did not change (*F_3, 11_* = 0.344, *p* = 0.794; Figure 5F). The oxidoreductase gene *CmGMC10* regulated the ROS level of the heat-acclimated larvae under heat stress.

### 2.6. Effects of the Exogenous Oxidant and Antioxidant on the Enzyme Activity

**Figure 6:**
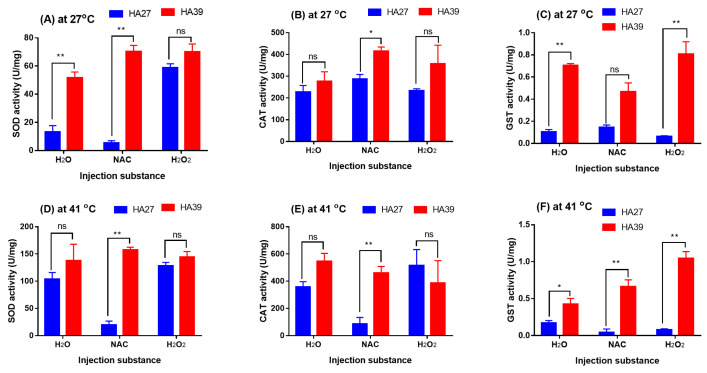
The activity of SOD, CAT, and GST in the HA27 and HA39 at 27 °C (**A**–**C**) and 41 °C of heat exposure for 2 h (**D**–**F**) after injection of antioxidant NAC and oxidant H_2_O_2_. * and ** indicate significant difference between HA27 and HA39 at *p* = 0.05 and *p* = 0.01 levels, respectively. ns: not significant.

Exogenous oxidant H_2_O_2_ induced a significant increase in the SOD activity in the HA27 larvae at 27 °C (*F_2, 6_* = 77.378, *p* < 0.001) but did not affect the HA39 (*F_2, 5_* = 4.843, *p* = 0.068; Figure 6A). The SOD activity of HA39 larvae was significantly higher than that of the HA27 at 27 °C when larvae were injected with H_2_O (t = 6.174, df = 4, *p* = 0.003) and NAC (t = 16.947, df = 3, *p* < 0.001), but the activity became similar when larvae were injected with H_2_O_2_ (t = 1.789, df = 4, *p* = 0.148; Figure 6A). Injection of NAC and H_2_O_2_ did not affect the CAT activity of HA27 (*F_2, 6_* = 2.204, *p* = 0.192) and HA39 larvae (*F_2, 5_* = 1.103, *p* = 0.401) at 27 °C (Figure 6B). The CAT activity in the HA27 and HA39 at 27 °C was similar when these larvae were injected with H_2_O (t = 0.923, df = 4, *p* = 0.408) and H_2_O_2_ (t = 1.466, df = 4, *p* = 0.217), but the activity in the HA39 was significantly higher than that in the HA27 when injected with NAC (t = 4.216, df = 3, *p* = 0.024; Figure 6B). Injection of H_2_O_2_ decreased the GST activity of HA27 larvae (*F_2, 6_* = 5.273, *p* = 0.048) but did not affect the HA39 (*F_2, 5_* = 3.891, *p* = 0.096) larvae at 27 °C (Figure 6C). The GST activity in the HA39 at 27 °C was significantly higher than that in the HA27 when they were injected with H_2_O (t = 22.176, df = 4, *p* < 0.001) and H_2_O_2_ (t = 6.599, df = 4, *p* = 0.022), but there was no difference when injected with NAC (t = 4.869, df = 3, *p* = 0.135; Figure 6C). When larvae were exposed to 41 °C for 2 h, the HA39 showed similar activities of SOD and CAT to the HA27 when they were injected with H_2_O and H_2_O_2_, but the activities were significantly lower in the HA27 than that in the HA39 when injected with NAC (SOD: t = 15.682, df = 4, *p* < 0.001; CAT: t = 5.432, df = 4, *p* = 0.006; Figure 6D,E). Under exposure to 41 °C, the GST activity in the HA39 was significantly higher than that in the HA27 when they were injected with H_2_O (t = 3.013, df = 4, *p* = 0.039), NAC (t = 5.914, df = 4, *p* = 0.004), and H_2_O_2_ (t = 13.589, df = 3, *p* = 0.001; Figure 6F). The heat-acclimated larvae exhibited higher levels of enzyme activity of SOD, CAT, or GST to confront NAC or H_2_O_2_ (Figure 6).

### 2.7. The Titer of H_2_O_2_ in the HA27 and HA39 Larvae under Heat Stress

**Figure 7:**
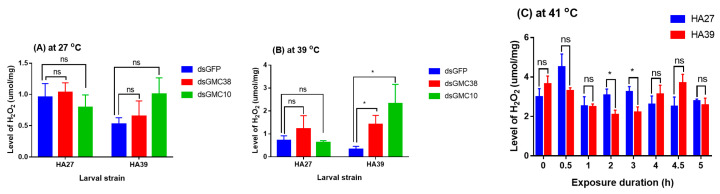
RNAi of *CmGMC10* or *CmGMC38* (**A**,**B**) and heat exposure to 41 °C (**C**) affect the levels of H_2_O_2_ in the HA27 and HA39 larvae. * indicates a significant difference between the HA27 and HA39. ns: not significant at *p* = 0.05 level.

At 27 °C, knockdown of *CmGMC38* or *CmGMC10* did not affect the level of H_2_O_2_ in the HA27 and HA39 larvae (Figure 7A), but at 39 °C for 3 h, knockdown of *CmGMC38* (t = 3.346, df = 5, *p* = 0.040) and *CmGMC10* (t = 3.915, df = 5, *p* = 0.022) increased the H_2_O_2_ level in the HA39 larvae, whereas it did not affect the HA27 (Figure 7B). Heat acclimation affected the accumulation of H_2_O_2_ in larvae exposed to a high temperature (Figure 7C). The titer of H_2_O_2_ in the HA27 larvae was significantly higher than that in the HA39 when they were exposed to 41 °C for 2 h (t = 2.509, df = 8, *p* = 0.036) and 3 h (t = 2.744, df = 8, *p* = 0.025), although there were no significant differences between these two strains when exposed to 41 °C for 0–1 h and 4–5 h (Figure 7). Heat acclimation decreased the accumulation of H_2_O_2_ under a certain degree of heat stress.

## 3. Discussion

Temperature plays an important role in the physiology and life history of insects [42,43]. Insects also show a certain degree of adaptability to temperature, including extreme temperature [41,44,45]. The responses of organisms to thermal change involve enzyme activity. A previous study has shown that differentially expressed genes between HA27 and HA39 of *C. medinalis* when exposed to 41 °C were enriched in the GO term of oxidoreductase activity [10]. Here, we found that two glucose dehydrogenase genes, *CmGMC10* and *CmGMC38*, were expressed differentially between the HA27 and HA39 under heat stress. The expression level of *CmGMC10* was significantly higher in the HA39 than in the HA27 under heat stress, suggesting this gene may be associated with heat acclimation. The comparison of transcriptomes showed that differentially expressed genes between thermo-sensitive and thermo-resistant strains of *B. mori* were significantly enriched in the GO terms, metabolic process, extracellular region, and serine-type peptidase activity when larvae were exposed to 35 °C [46]. In *Tribolium castaneum*, acclimation to a sublethal high temperature enhanced the heat tolerance of adults, and the upregulated genes between different temperature-acclimated adults exposed to 50 °C for 25 min were enriched in these categories, the terms of membrane part, metabolic process, and catalytic activity [47]. The wa`rm-acclimated rainbow trout *Salmo gairdneri* would decrease the GDH activity in the liver when the temperature decreased, but the long-time cold-acclimated did not [48], suggesting that changing temperature affects glucose dehydrogenase. Temperature significantly affected the enzyme activities and gene expression of hepatic glucokinase, glucose-6-phosphatase, and glucose-6-phosphate dehydrogenase in the tilapia *Oreochromis niloticus* [49]. In this study, a glucose dehydrogenase gene, *CmGMC10*, was more highly expressed in the body wall of the heat-acclimated larvae when confronted with heat stress, implying that the glucose dehydrogenase gene may mediate heat acclimation.

The result of RNAi further confirmed that the *CmGMC10* regulated heat acclimation of the rice leaf folder larvae. Although the interference efficiency when using dsRNA or dsRNA+dsCmdsRNase was not very high, the heat tolerance of larvae injected with dsCmGMC10 or dsCmGMC38 was significantly decreased. RNAi of *CmGMC10* led to a significant reduction in the survival rate of the HA39 larvae under heat stress. The RNAi using dsRNA is not a stable method to unfold the gene function of lepidopteran insects, but it is an alternative method now prior to the establishment of a simple and economical gene-editing method such as CRISPR/Cas9 [28,30,50]. In this study, we injected dsRNA or dsRNA+dsCmdsRNase to knock down the *CmGMC* gene, and similar results were attained, suggesting that the RNAi method was relatively effective for these two genes. RNAi of *CmGMC10* resulted in a decrease in the larval survival rate and an increase in the ROS level of HA39 larvae under heat stress, but injection of dsCmGMC10 or dsCmGMC38 did not result in a change in ROS in the HA27. The result indicated that the *CmGMC10* was related to the ROS level of larvae under heat stress.

**Figure 8:**
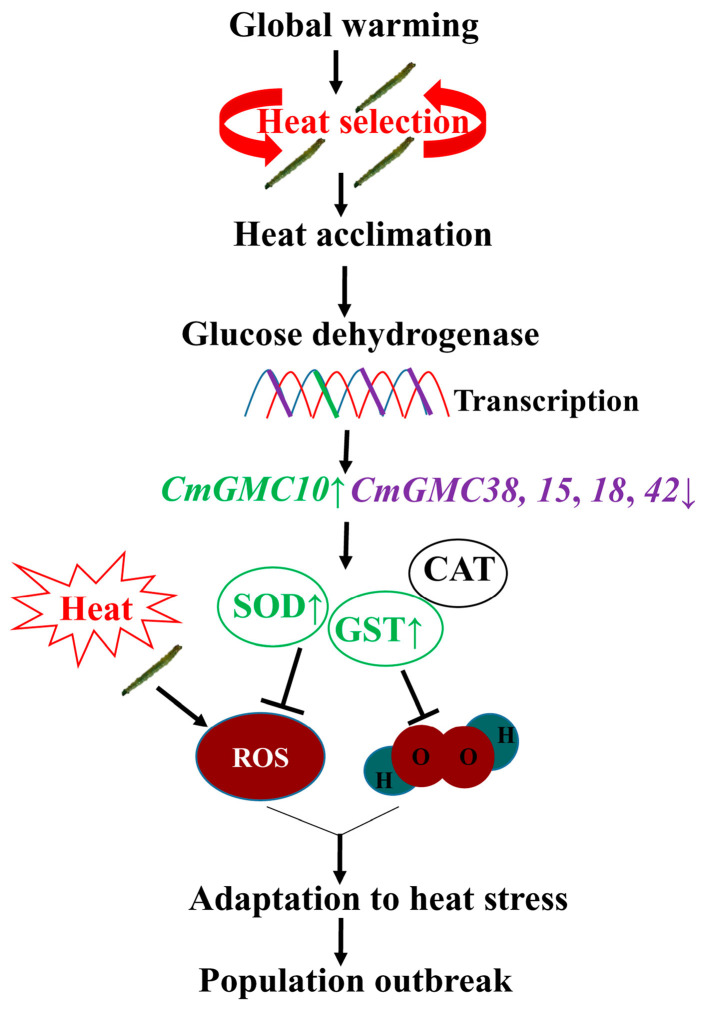
A mode of glucose dehydrogenase-mediated heat acclimation of larvae to deal with global warming. **↑** means the up-regulated gene or enzyme activity. **↓** means the down-regulated gene.

The *CmGMC10* and *CmGMC38* belong to the oxidoreductase gene, which regulates the activity of antioxidant enzymes, such as SOD, CAT, and GST. The CAT, GST, and total antioxidant capacity were significantly increased in the ladybeetle *Propylaea japonica* when exposed to 35–43 °C, and the SOD was also increased when the temperature was above 37 °C [51]. An increase in temperature to 28 °C elevated the enzyme activity of SOD and CAT in aphids, and the antioxidant enzyme system protected aphids from high temperatures [52]. In the leaf folder larvae, the SOD and GST in the HA39 were significantly higher than that in the HA27 at 27 °C, and the GST was still higher at 41 °C. The relative expression level of *CmGMC10* of the HA39 was higher than that of the HA27 at 41 °C. Overexpression of *CmGMC10* in the heat-acclimated larvae enhanced enzyme activity and antioxidant defenses, and consequently conferred higher heat tolerance to the heat-acclimated larvae. SOD can convert superoxide anion into oxygen and H_2_O_2_, and GST can remove the hydroperoxide from cells [53,54,55]. The level of H_2_O_2_ in the HA39 larvae was lower than that in the HA27 when exposed to 41 °C for 2 or 3 h, and the ROS level increased in the HA39 larvae under heat stress when their *CmGMC10* was knocked down, but these results were not found in the HA27. These results further confirmed that heat acclimation was mediated by the highly expressed glucose dehydrogenase gene *CmGMC10*, which reduced the level of oxidative stress in larvae under high temperatures (Figure 8).

Global warming is a threat to insects [56,57], while it may act as a selection stress driving the evolution of heat adaptation. Multigenerational selection under a high temperature increased heat tolerance to certain higher heat stress in the rice leaf folder [8,10], and this heat tolerance had transgenerational effects [41]. The slowly increasing ambient temperature from year to year may be similar to multigenerational selection and induce the heat acclimation of insects. In this study, we found that heat acclimation was mediated by the overexpression of a glucose dehydrogenase gene, suggesting a phenotypic plasticity (Figure 8). Transgenerational plasticity affects the heat tolerance of the offspring of the exposed generation [58,59,60]. Therefore, the population of rice leaf folder can adapt to increasing heat stress, and population outbreak will continue under global warming conditions.

## 4. Materials and Methods

### 4.1. Establishment of the Heat-Acclimated Strain

A population of about 600 larvae of *C. medinalis* was collected from a rice field in Nanjing, China in 2010. They were reared in the laboratory using wheat seedlings at 27 °C, 60% Rh, and a photoperiod of 14L:10D [61]. From the population, a part of the third instar larvae was exposed to 39 °C for 3 h per day at 11:00 a.m. (local time) for 3 d, and then all heat-treated larvae were reared at 27 °C. The same heat treatment was performed in the following generations. After more than 5 generations, the heat-treated population was considered as the heat-acclimated strain, HA39 [8,10,28]. The other part of the larvae was persistently reared at 27 °C and considered as the unacclimated strain (HA27).

### 4.2. Heat Tolerance of Larvae

Thirty third instar larvae from the HA27 (reared for about 123 generations at 27 °C in the laboratory) and HA39 (heat acclimated to 39 °C for about 54 generations) were transferred into a plastic cup with 30 wheat seedlings. Those larvae were exposed to 43 °C for 0.5 and 1 h, and then their survival was examined. All the surviving larvae were transferred into a 27 °C climate chamber and reared using new wheat seedlings. After 2 and 4 days, the survival of all larvae was examined again. Therefore, survival rates of tested larvae at the 0, 2nd, and 4th day after heat exposure were computed. The experiments were replicated three times for the HA27 and HA39.

### 4.3. Examination of the Relative Expression Level of Oxidoreductase Gene

In the transcriptome analysis, 21 differentially expressed putative genes between HA27 and HA39 after exposure to 41 °C for 1 h were enriched in the oxidoreductase activity GO term [10]. The relative expression levels of these putative genes of the HA27 (reared for 96 generations) and HA39 (heat-acclimated for 27 generations) were examined using the qPCR method. Ten third instar larvae of the HA27 and HA39 reared on a cup of wheat seedlings were exposed to 41 °C for 1 h in a climate chamber, and then three larvae were collected as a sample and quickly frozen in liquid nitrogen. Three samples were collected for the HA27 and HA39 and maintained at −80 °C until being used to extract RNA for the qPCR. We found 4 out of 21 putative genes were expressed differentially, including one upregulated and three downregulated putative genes in the HA39, compared to HA27. Therefore, we selected the one upregulated 121117 and one downregulated 71513 genes to examine the spatiotemporal expression in the HA27 and HA39 under different heat exposure durations of 0, 0.5, 1, 2, and 3 h to 39 °C and in different tissues or parts of the third instar larvae at 27 °C. Three replicates were performed, but one sample in the HA39 that was exposed for 2 h failed the qPCR for the *CmGMC38* gene. These tissue samples from the HA27 (reared for 98 generations) and HA39 (heat acclimated for 29 generations) were collected from the head, silk gland, epidermis, foregut, midgut, and Malpighian tubules. Each sample was dissected from 10 third instar larvae and stored in a 1.5 mL centrifuge tube at −80 °C after quick freezing in liquid nitrogen. Three samples for each tissue were collected and examined, except the foregut of HA27, in which one sample failed qPCR.

RNA of the sample was extracted using the TRI-ZOL method (Takara, Dalian, China). The cDNA synthesis was performed using a PrimeScriptTM RT Reagent Kit with gDNA Eraser (Perfect Real Time) (Takara, Dalian, China) according to the instructions. The synthesized cDNA was stored at −80 °C for qPCR. The test kit for qPCR was a TB GREEN Premix Ex Taq Kit (Takara, Dalian, China). qPCR was performed in the ABI 7500 (Applied Biosystems, Carlsbad, CA, USA) using these primers listed in Table S1. Actin and RPs15 were used as the internal reference genes because they were expressed stably in the larvae under heat treatment (Figure S1). The relative expression levels of two oxidoreductase genes were calculated using the 2^−ΔΔCt^ method based on two reference genes [62]. Three technical replicates were performed for each RNA sample.

### 4.4. Clone of Two Oxidoreductase Activity Genes

The full length of the gene 71513 and a partial sequence of 121117 were cloned using the SMARTer RACE 5 ‘/3’ Kit (Takara, Dalian, China) based on the primers in Table S2. The gene clone method is shown in the supplementary information. Based on the sequence (Figure S2) and the genome of the rice leaf folder [63], two oxidoreductase activity genes, 121117 and 71513, were identified as *CmGMC10* and *CmGMC38*, respectively (Figure 1).

### 4.5. Knockdown of Two Oxidoreductase Activity Genes

The 300-400 bp dsRNA of *CmGMC10* and *CmGMC38* was designed using the online software E-RNAi Design version 3.2 (https://www.dkfz.de/signaling/e-rnai3/, accessed on 8 May 2020). The synthesis of dsRNA was performed according to instructions of the T7RiboMAXTM Express RNAi System (Promega, Madison, WI, USA) based on primers in Table S3. Two dsRNAs were designed for the *CmGMC38* and their mixture reagents were used for RNAi. Only one dsRNA was designed for the *CmGMC10* and *GFP*. The sequenced gel recycling products were used as templates to synthesize dsRNA. The reaction system included 10 μL RiboMAXTM Express T7 2x Buffer, 1–8 μL templates, 0–7 μL nuclease-free water, and 2 μL enzyme Mix T7 Express. The integrity of the synthesized dsRNA was detected using 1.2% agarose gel electrophoresis, and its concentration and purity were measured by spectrophotometer. All dsRNA samples were stored at −80 °C. It has been known that dsRNA degradation enzymes in lepidopteran insects lead to a low interference efficiency of RNAi [64] and the dsCmdsRNase increased the interference efficiency of the chitin synthase gene of *C. medinalis* (*CmCHS*) by 27.17% [34]. Therefore, we synthesized the dsCmdsRNase to improve the interference efficiency. The dsCmdsRNase was injected after mixing with the dsRNA of a target gene (1:1).

To determine the optimal interference efficiency of RNAi, the dsCmGMC38-1 and dsCmGMC38-2 were mixed (1:1) as dsCmGMC38. The third instar larvae of HA27 (reared for about 119 generations) and HA39 (heat acclimated for 50 generations) were collected, and 100, 200, 300, 400, and 500 µg of dsCmGMC38 were injected at the 6–8th segment of the abdomen under the Micro4/nanoliter microinjection system (World Precision Instruments, Sarasota, FL, USA). Forty larvae were injected for each dose of dsCmGMC38. After injection, the larvae were reared using fresh wheat seedlings at 27 °C for 24, 48, and 72 h, and then three larvae were collected to extract RNA for the examination of the relative expression level of *CmGMC38* using qPCR. Based on the relative expression levels, we found that the interference efficiency was the highest 48 h after injection of 400 µg of dsRNA (Figure S3). Therefore, in the RNAi experiments, 400 µg of dsRNA was used. The third instar larvae from both the HA27 (reared for 144 generations) and HA39 (heat acclimated for 75 generations) were divided into three groups. One group injected dsGFP (CK), another injected dsCmGMC38 or dsCmGMC10, and the other injected dsCmdsRNase+dsCmGMC38 or +dsCmGMC10. Each group injected 30 larvae. Forty-eight hours after injection, the expression level of the *CmGMC38* or *CmGMC10* genes of 3 larvae was detected using qPCR to determine the interference efficiency, and the other injected larvae were transferred into tubes (25–30 larvae per tube) for exposure to 39 °C in a water bath for 3 h. After the exposure, the survival rate of the larvae was examined, and then all the heat-exposed larvae were reared on the wheat seedlings at 27 °C. The survival rates of larvae were surveyed at 2, 4, and 6 days after heat exposure. Injection of the larvae of HA27 and HA39 with each dsRNA was performed in three or four replicates.

### 4.6. Detection of Enzyme Activity and ROS Level

After dsRNA injection and heat exposure to 39 °C for 3h, three larvae were collected from the HA27 (reared for 144 generations) and HA39 (heat acclimated for 75 generations) strains reared in a cup of wheat seedlings as a sample. Three or four replicates were performed for the HA27 and five to seven replicates were performed for the HA39. Larval samples for each treatment of RNAi were collected to examine the enzyme activity. One milliliter of PBS was added to each sample and then ground. The supernatant after centrifugation at 4 °C, 12,000 rpm for 15 min was the crude enzyme solution to measure the content of protein, the activity of SOD, CAT, and GST, and total antioxidant capacity (T-AOC) using the BCA Protein (Beyotime, Shanghai, China), SOD, CAT, GST, and T-AOC Assay Kits (Solarbio, Beijing, China), respectively. When measuring the level of ROS after RNAi and heat exposure, three larvae were ground using 1 mL cell lysis buffer (Beyotime, China), and then centrifuged at 12,000 rpm at 4 °C for 15 min. The supernatant was used to measure the protein content and ROS level using the BCA Protein and Reactive Oxygen Species Assay Kits (Beyotime, China), respectively. The levels of ROS in the HA27 (reared for 134 generations) and HA39 (heat acclimated for 65 generations) larvae exposed to 41 °C for 0, 0.5, 1, and 2 h were also examined using the same method as above, and three or four replicates were performed.

### 4.7. Enzyme Activity of Larvae Injected with Exogenous Oxidant and Antioxidant

The antioxidant N-Acetyl-L-cysteine (NAC) can inhibit the ROS [65] and the oxidant H_2_O_2_ can increase the ROS level in insects [66]. To explore the relationship of oxidant and antioxidant with enzyme activity, 200 nL exogenous oxidant H_2_O_2_ (75 mg/mL) or antioxidant NAC (12.5 mg/mL) were injected into the third instar larva of HA27 (reared for 137 generations) and HA39 (heat acclimated for 68 generations) at the 6–8th segment of the abdomen using the Micro4/nanoliter microinjection system (World Precision Instruments, Sarasota, FL, USA), and injection of 200 nL water was the control. Sixty to ninety larvae were injected for each of the exogenous substances and then reared using wheat seedlings at 27 °C. After 24 h, 15–30 injected larvae on a cup of wheat seedlings were exposed to 41 °C for 2 h, and the 30 injected larvae kept at 27 °C were used as control. After heat treatment, 3 larvae from a cup of wheat seedlings were collected as a sample for examination of the SOD, CAT, and GST activity. Three replicates were performed for each combination of injection and heat treatment, but a sample failed to be examined for each the HA39 larvae injected with NAC at 27 °C and those injected with H_2_O_2_ at 41 °C. 

### 4.8. Examination of the H_2_O_2_ Level in Larvae

The H_2_O_2_ levels in the third instar larvae of HA27 (reared for 144 generations) and HA39 (heat acclimated for 75 generations) injected with dsCmGMC10 and dsCmGMC38 after 48 h were detected when they were exposed to 27 °C and 39 °C for 3 h. Four replicates were performed, but one replicate failed in the HA39 knockdown of *CmGMC10* or *CmGMC38*.

Twenty third instar larvae of the HA27 and HA39 reared on a cup of wheat seedlings were transferred into tubes and exposed to 41 °C for 0, 0.5, 1, 2, 3, 4, 4.5, and 5 h, respectively. Three surviving larvae were transferred into a 1.5 mL centrifuge tube and then transferred to a refrigerator at −80 °C for examination of the H_2_O_2_ level after rapid freezing in liquid nitrogen. Five replicates were performed. Each sample had 300 µL acetone added and was homogenized in ice bath, centrifuged at 8000 rpm at 4 °C for 10 min, and the supernatant was placed on ice for examination using the BCA protein detection kit (Beyotime, China) and H_2_O_2_ Assay Kits (Cominbio, Suzhou, China) to examine the protein concentration and H_2_O_2_ level, respectively. 

### 4.9. Data Analysis

The standard statistical analyses, the Student’s *t*-test, followed by the Bonferroni correction and ANOVA, followed by post hoc Tukey’s test, were used to analyze the data except those specially mentioned below. The log transformation for the relative expression levels of genes after RNAi was used before the statistical analysis because the data did not meet the normal distribution. The survival rates of larvae with knocked down *CmGMC10* or *CmGMC38* after heat exposure were analyzed using the repeated-measures ANOVA in the GLM model, and the survival rates measured at 0, 2, 4, and 6 d after exposure were considered as repeated measurements. All analyses were performed in IBM SPSS Statistics software V25.

## Data Availability

The data underlying all the results presented in the paper have been archived in figshare: 10.6084/m9.figshare.22546621 [67].

## Supplementary Materials

The following supporting information can be downloaded at: https://www.mdpi.com/article/10.3390/ijms241210146/s1.
