# Supplementary material for: Glucose Dehydrogenases-Mediated Acclimation of an Important Rice Pest to Global Warming
Source: Int J Mol Sci. 2023 Jun 14;24(12):10146. doi: 10.3390/ijms241210146 (PMC10298946; doi:10.3390/ijms241210146)
Supplement: Supplementary file 1 [file ijms-24-10146-s001.zip › ijms-2413148-supplementary.pdf]

## Methods

*Screen of internal reference genes*

Five reference genes, *EF1-a*, *18S*, *TUB*, *actin*, and *RPs15*, which were stable in the rice leaf folder at 27 °C, were selected to check the stability under heat shock. The 3rd larvae from the HA27 and HA39 were exposed to 41 °C for 1h and then collected the samples. The HA27 at 27 °C was control. The expression levels of five internal reference genes in all samples were detected by qPCR after reverse transcription, and the CT values were analyzed using geNorm software to calculate the average expression stability (M). The results showed that *actin* and *RPs15* were the most stable reference genes with the lowest M in the *C. medinalis* exposed to 41 °C (Fig S1), so these two genes were chose as internal reference genes in this study.

*Clone of 71513 and 121117 genes*

The full length of the gene was obtained using SMARTer RACE 5 '/3' Kit (Takara, Dalian, China). The partial sequence of 71513 was searched based on transcriptome data (Quan et al., 2020). Primer Premier 5 software was used to design primers and clone the gene. The 5' and 3' ends were amplified according to the instructions, and nestler PCR was used for amplification. UMP and specific primers 71513-1-5 'and 71513-1-3' were used for the first round of PCR. In the second round of PCR, NUP and specific primers 71513-2-5' and 71513-2-3' were used (Table S2). 1.2% agarose gel

electrophoresis was used to detect whether the length of PCR products was consistent with the target fragment. If the length was consistent, the PCR products were recovered and purified (TransGen Biotech, Beijing, China). Peasy-t3 Cloning Kit (TransGen Biotech, Beijing, China) was used to connect the purified PCR product to the vector and converted it to the plasmid of the Trans-T1 Phage Resistant Chemically Cell (TransGen Biotech, Beijing, China), and then sequenced (TsingKe, China). The 5' end sequences and 3' end sequences were matched with DNAMAN6.0 software. The sequences were analyzed using the NCBI (https://www.ncbi.nlm.nih.gov/), Superfamily (https://supfam.org/SUPERFAMILY/index.html), Pfam (http://pfam.xfam.org/), ExPASy (http://web.expasy.org/compute_pi/) online software. Protein sequence alignment was performed in online software STRING (https://cn.string-db.org/cgi/input?sessionId=UjInM0YjECUF). Download 26 similar sequences from NCBI and STRING, and the phylogenetic tree was constructed by MEGA-X software using neighbor-joining (NJ) method. Outgroup is a gene selected from the *C. medinalis* transcriptome.

The partial sequence of 121117 was searched in the transcriptome database (Quan et al., 2020), and 4 pairs of primers were designed by Primer Premier 5 software. After PCR amplification, electrophoresis was performed, and only the fourth pair of primers (Table S2) was specifically amplified. A 663 bp sequence was obtained after sequencing (TsingKe, China), and the full length was cloned by Race method. But the

sequence of 5' end and 3' end was not obtained, so only a part of CDs was used for sequence analysis. The analysis method was the same as sequence 71513.

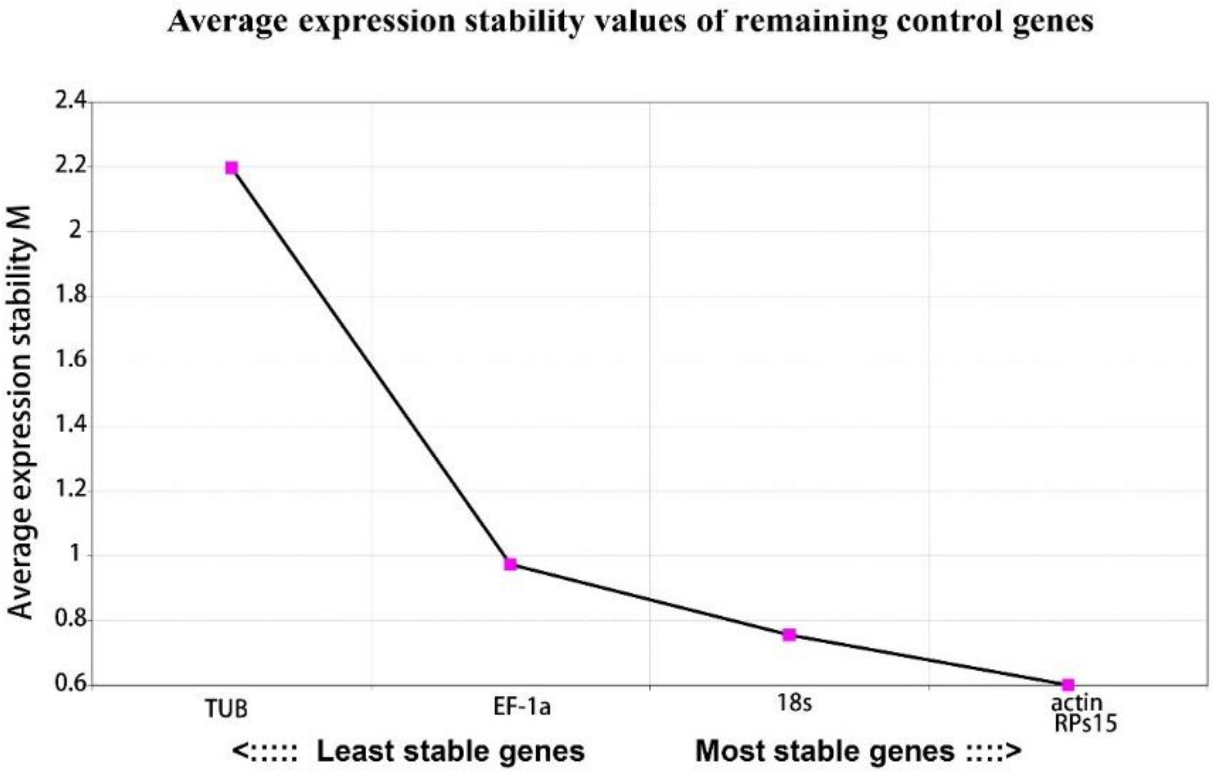


**Figure S1** Screening of internal reference genes

(A) >121117 *CmGMC10*

TTCGGGCTAATCGCTAGTTTGAACTATAAAACAGCCGACTTTATTTCCCTGTAACACAGGC
CCTGTGGCCTCTATCAGGCATAACCTTATCCTAATTTTACAGCCGGACCTCAATACTGTTTT
CTAGTGTTAAACCCTTTGTTATAAAGTGCATGAAAGAAATAAATGTCTTTTTTTTATTATGGT
TGGACTTGTGTTCACTAATAGTAGGTACAATAACTAAATTAGTTTAAAATATAAAAGATTAG
GTAAACCAATGAGATTTATTCAATAATAAATAAAGCCTTTATTGATAAAATAACAGCCGAA
TTACACTAAATTGTTAAAATACTTAGCATGTTTAATAACACGTGTTAAAATGAAGGAACGA
AACATAAGATATGTACTGAATTATACTATAGTAATTATAACACGAACTAGATTATTTGATTCG
CTATTAACATTGACCTTCTACAGGTATGCCATAGTCTCTTTTGATCAAACTGGAAGCTCTCT
CCCCAATCATGATGACTGGCCCATTAGTGTTTCCACTCACATGAGATGGCATCACACTCGC
GTCTGCCACTCGCAGTTTGTTTATAGAGTGAACCTTCAATCTACTACTTACTACAGACGTCT
TCATATCATCACCCATTCTAGCAGTCCCCACTGGATGATAAACAG

(B) >71513 *CmGMC38*

AAGCAGTGGTATCAACGCAGAGTACATGGGAAGCAGTGGTATCAACGCAGAGTACATGG
GGCAGTGGTATCAACGCAGAGTACATGGGGGCCACTCCGCAAGTTCGTACCTTCTTCTTA
ATTTGGCTACGGGACCTTAAAAAGTGAAAAACTTTTACCAGTAACATCCGTCATGACGTC
AACAGTGGAAGCTGTAGTGAGCTCTGTACGGGCCATACAGGGTGCCTTACTAGTGATTGC
AGGGCTACAGCTGACAGGGTACATGTATCCACAGTCCACTACCGTTACAAATGGAGCCTC
ATATGATTTTATTATATGCGGAGCCGGCACTGGAGGGAGTGTCATTGCGAACAGACTGACA
GAAATCCCCAACATTAATGTACTCCTTATTGAAGCTGGGGATGATCCTAGAATAGAATCAT
TACTTCCAGGTTTGCTGACGCTACAGCCTCTCACGGAACAGGACTTCAACTTCACTTCTGA
AGACGATGGTAGAACAGGACAGTACCTCAAAGACAGGGTAGTGGGTATGTCTCAAGGGA
AAATGCTGGGAGGTAGCAGTAGCTTGAACCATCTCATTCATGCAAGAGGAAACGCTAGGG
ACTACCAAAGGTGGGCAGACGCAGCTCAAGAcGAATCCTGGAACTTTGACAATCTCATGC
AGTATTTCATTAAGAGTGAGAAAGTTGAGGATGAAGACATCCTCAAATCTAAACTTGGGA
AATACCACGGAACAGAAGGCTATTTAAGAGTATCTACTCAACGCAGTGACGAGAACATAC
CAATTTTTGAGGCTTTCGCTGAATTAGACCACGAGAATGTTTTAACAGCTAACACTCCAGA
ATTTACAGTCGGTATTACAGAACCTTTGCTGAACATTGCTGATGGCAAAAGACAAAGTACT
GCAGAATATTTGAGACCACTCTCGGACCGTCCTAATTTCCATATAATGAAAAATACACACG
TGACTAAAGTATTGTTTGATGATGACAGGAATGCTATTGGCGTGGAAGCTGTTACAGCTGG
TGGAGAAACTGTTACGATCAACGCAAATGTTGAGGTTATTTTAGCAGCCGGTGCTTTTGTG
ACTCCTCAACTACTTATGCTTTCCGGAGTTGGCCCCAAAGATCATTTAGAGTCCTTCAATAT
TGATGTTATCTCAGATTTGCCTGTTGGCAAGAACCTCCAAGATCACGTAGATCCAGTAGTT
ATCCATGCGTTACAAGAAGGTGAAGCTCCTACTGCTCCAGCCAATCCTCACGAATATCCA
GTACCAACTACCGTTGCTTACACTGCTCTAGACATGAATCAAGGGTATCCTGACTACCAAA
CGATAAACTTGCTCTTCCCTCCAGACTCCACAGGTTTGATTCAGTTCTGCTCTTTAGTCTTT
GCTTATAACGACGAGATTTGTCAAAAATGGTTTGATGGTGGTAAAGGGCGGTATACTTTAT
TCACCTGCCATAATCTTATGCAGCCGTATTCAACTGGAGAAGTTCTCCTCCGTAGTGCAGA
CCCGAAAGACCCTCCTATCATCCACCACGGAATCTTCTCAAACGAAACTGATCTACACAA
CATGGCGTTGTACCTTAAAGACTTCGCTCGCTTCGGCAAAACGTCGTATATGAAAAATGTT
GGAGGTTTCTTGATTGATCTGGAATTGGAAGAATGCAAAGGTTTATCAAAGGATTCTTATG

AATACTGGAGATGTTACGCCCTGAGTATGTCGGCTACTTTATGGCACTACAGCAGCACAGC
GTCTATGGGACCGGTTCTGGACAGCCACTTGAGAGTTAAAGGAGTGAACAGGCTGAGGG
TTGCCGATTCTAGTGCCATGCCTAATACCGTAAGTGGTAACCCAATGGCAGCGGTGGTGGC
GCTTGCAGAAAAAGCAGCAGATTTGATTAAGAAAGATAATGGTGCAAGTCCCAGAAGTA
GTAATTAAATTTCTTGTTGAAGATCGTATTTAAGTATAATTGTTTTTTTGTGCTTCCTTATTAC
TTTGTTCAATGAAACCATTTGTTATTAAAAAAAAATATAAGGAGATGTATTCTGTAGACGC
CGGTAAACCTAATATACAAACGAAACGAAACACAAGCGCAGTGCACTCAAAGCCTACTG
CCATCTATTGGTAGACAGTGGAATTATTATTACGAAAAGAAGTGTTAAAAAATAGATTGGG
TTGACATCATTAATAGTGCATTAGCATAAGTTATCCATTAAATGTTTTTTAAAAAAAAAAAA
AAAAAAAAAAAAAAAAAAAAAAAAAAAAAAAAA

**Figure S2** The partial sequence of *CmGMC10* (A) and the full length of *CmGMC38* (B) of the rice leaf folder larvae cloned in this study.

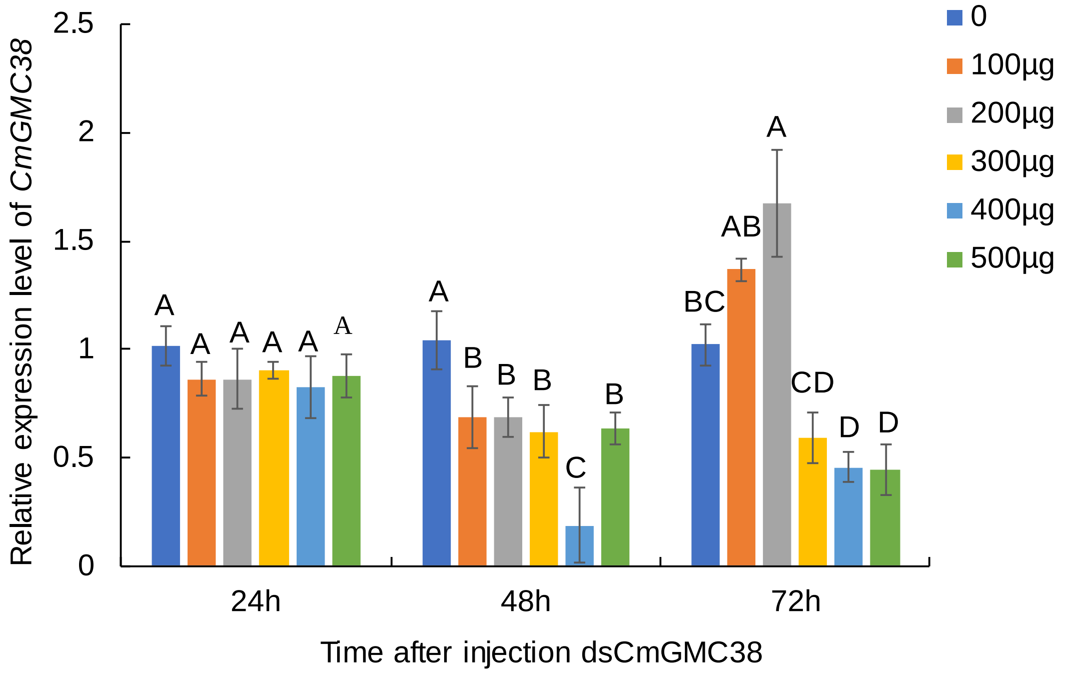


**Figure S3** Interference efficiency of *CmGMC38* using injection of dsCmGMC38 with different dosages (0-500 ug). The different letters indicate significant difference among doses of dsCmGMC38.

**Table S1** Primers used in RT-qPCR

| Primer | Sequence（5′-3′） |
|---|---|
| *actin*-F | ATGGTCGGCATGGGACAG |
| *actin*-R | GAGTTCATTGTAGAAGGTGT |
| *RPs15*-F | ACGTACCCGCTTACAAACCC |
| *RPs15*-R | TGACCAAGGTGAGCAACAGAG |
| *18s-F* | CCGATTGAATGATTTAGTGAGGTC |
| *18s-R* | CCTACGGAAACCTTGTTACGAC |
| *TUB-F* | ACCTACGGCGACTTGAACCA |
| *TUB-R* | AGAAATGAAGACGAGGGAACG |
| *EF-1a-F* | CCTATTTCTGGATGGAATGGTG |
| *EF-1a-R* | GAGGCTTGTCAAGTGGTCGA |
| 71513-F | TATGCAGCCGTATTCAACTGG |
| 71513-R | AGCGAGCGAAGTCTTTAAGGTA |
| 121117-F | GTCTGCCACTCGCAGTTTGT |
| 121117-R | GGGACTGCTAGAATGGGTGAT |
| 118955-F | TACAACTACGTGTCGGTGGC |
| 118955-R | TTTCCCCCTTGACGCTCTTC |
| 99097-F | TACAACTACGTGTCGGTGGC |
| 99097-R | TTTCCCCCTTGACGCTCTTC |
| 75011-F | TTGAGGTGCTATGGGCGATG |
| 75011-R | ATAACGCGGAGTCCCTTGAC |
| 54999-F | GGTCGCACGCTGTTCATTAC |
| 54999-R | CGCAGCGACAACCACATTAG |
| 27598-F | TGTGGAAGTGCCACCTAACC |
| 27598-R | TTTTCCTGTACTCCCGAGCC |
| 87221-F | CCAGGATTCGTCTTGGGCTG |
| 87221-R | CTGGGAGGTAGCAGTAGCTTG |
| 66582-F | GGAGGGAGTGTCATTGCGAA |
| 66582-R | GTAGCGTCAGCAAACCTGGA |
| 21996-F | CTGCAGACTATCGCGTCACT |
| 21996-R | TCTGAAAGCCTGGACGCTAC |
| 67877-F | ATTTGTTCCCCGTCCCCATC |
| 67877-R | TGAGCTGTGTTATCGGCAGG |
| 85040-F | TTTGCTGCTGACTCCATCGT |
| 85040-R | TTATGTGGCAGCAAGCGGTA |
| 48050-F | CAACACAGCGAGGATGGGAA |
| 48050-R | CCACTGATGGAGTTCGGCAT |
| 141193-F | CACCTGCGATGGGTACTGTT |
| 141193-R | TAGCATTGTCCGTCGCCTTT |
| 87222-F | GGAGGGAGTGTCATTGCGAA |
| 87222-R | GTAGCGTCAGCAAACCTGGA |
| 74602-F | GGAGGGAGTGTCATTGCGAA |
| 74602-R | GTAGCGTCAGCAAACCTGGA |
| 81817-F | ATTTGTTCCCCGTCCCCATC |
| 81817-R | TGAGCTGTGTTATCGGCAGG |
| 102013-F | TGCTAGTATCATGCCCACCG |

| | |
|---|---|
| 102013-R | TCACCATGTCAGCTGCTTTCT |
| 82425-F | ATTTGTTCCCCGTCCCCATC |
| 82425-R | TGAGCTGTGTTATCGGCAGG |
| 73862-F | TCCGTGTGAAGGGTGTCAAG |
| 73862-R | CATGATGGTTGGCGCGTTAG |
| 121220-F | CTTGGGTACGTGGAAACCGA |
| 121220-R | AGCTTGCTTCCATCCGTGAA |

**Table S2** Primers used to clone full-length of 71513 and 121117

| Primer | Sequence（5′-3′） |
|---|---|
| 71513-1-5′ | GCGTTGATCGTAACAGTTTCTCCACCAG |
| 71513-1-3′ | GGCAGACGCAGCCCAAGACGAATC |
| 71513-2-5′ | CCCAGCATTTTCCCTTGAGACATACCCA |
| 71513-2-3′ | AGACCCGAAAGACCCTCCTATCATCCA |
| UPM | Long: CTAATACGACTCACTATAGGGCAAGCAGTGGTATC AACGCAGAGT<br>Short: CTAATACGACTCACTATAGGGC |
| NUP | CTAATACGACTCACTATAGGGC |
| 121117-F | TTCGGGCTAATCGCTAGTTTG |
| 121117-R | GTCCCCACTGGATGATAAACAG |

**Table S3** Primers used to synthesize the dsRNA

| Primer | Squence(5′-3′) |
|---|---|
| *dsCmGMC38*-1-F | taatacgactcactatagggGCAGATGCAGCTCAAGATGA |
| *dsCmGMC38*-1-R | taatacgactcactatagggCAGCTTCCACACCAATAGCA |
| *dsCmGMC38*-2-F | taatacgactcactatagggACTGATTGAAGCTGGGGATG |
| *dsCmGMC38*-2-R | taatacgactcactatagggATGTTCTCGTCACTGCGTTG |
| *dsCmGMC10*-F | taatacgactcactatagggCAGCCGGACCTCAATACTGT |
| *dsCmGMC10*-R | taatacgactcactatagggGGGGAGAGAGCTTCCAGTTT |
| *dsCmRNase*-F | taatacgactcactatagggCGACAGGAATCGTCTTGAAG |
| *dsCmRNase*-R | taatacgactcactatagggAGGCTATACGAGCACGGAGGT |
| *dsGFP*-F | taatacgactcactatagggGCCAACACTTGTCACTACTT |
| *dsGFP*-R | taatacgactcactatagggGGAGTATTTTGTTGATAATGGTCTG |
